# Assessment of Fatty Acid-Specific Lipolysis by In Vitro Digestion and GC-FID

**DOI:** 10.3390/nu13113889

**Published:** 2021-10-29

**Authors:** Judit Tormási, László Abrankó

**Affiliations:** Department of Food Chemistry and Analytical Chemistry, Institute of Food Science and Technology, Hungarian University of Agriculture and Life Sciences (MATE), 1118 Budapest, Hungary; tormasi.judit@uni-mate.hu

**Keywords:** bioaccessible fatty acid content, Infogest digestion, Bligh and Dyer method, FAME, GC-FID

## Abstract

The nutritional relevance of food compositional data could be improved by taking the bioaccessibility of these constituents into account. A lack of routine methods to assess the bioaccessibility of fatty acids (FAs) in food is one of the limiting factors of doing so. An analytical protocol is proposed for routine assessment of the extent of lipolysis via in vitro digestion simulation methods in food products. The established method provides specific information on each FA individually. Steps of the protocol including the Bligh and Dyer chloroform/methanol/water extraction of esterified and free FAs from in vitro digesta, methyl ester derivatization, and GC-FID analysis were specifically tailored to help routine work and were harmonized with the Infogest in vitro digestion simulation protocol (both v1.0 and v2.0). The method was applied to assess the degree of FA-specific lipolysis in a baked fish (carp) meal and the results showed that the FA composition of the original food significantly differed from that of the distribution of FFAs in the digesta. The use of gastric lipase (in Infogest v2.0 protocol) increased total FA release by 9.5% and its specific impact on palmitic acid was the most prominent.

## 1. Introduction

Fats and oils are major nutrients consumed in a wide variety as single foods (like butter or margarine) and as part of food products or meals. Dietary fats and oils are largely comprised of triacylglycerides (TAGs), which are esters formed by one glycerol molecule and three fatty acids (FAs) [1]. The FA composition and quantity of consumed fats and oils are of great nutritional importance since essential FAs are supplied in this way via diet, whereas the consumption of saturated FAs is associated with an increased risk of cardiovascular diseases [2]. Instead of focusing on the amount of FAs in the ingested food, a more relevant picture can be obtained if the digestibility of FAs is also considered. Digestibility studies can provide information on the bioaccessibility of the nutrients of interest. The bioaccessible fraction of an ingested component is the part that is released from ingested food matrix during digestion and in the intestinal lumen in a suitable form for absorption [3].

Fat digestion starts in the stomach via acid-resistant gastric lipase; however, the majority of fat digestion occurs in the small intestine via pancreatic lipase [4,5]. After emulsification and enzymatic hydrolysis, TAGs are converted to free FAs (FFAs) and monoacylglycerides (MAGs). The FFAs and MAGs in the small bowel could be subjected to active transport through the epithelial wall into the bloodstream [6,7,8]. The bioaccessibility of triacylglycerides depends on the composition and structure of the food matrix, the presence of other components (bioactive, chemical, or toxic compounds), and is also influenced by processing, storage, and digestion conditions [1].

Measurement of bioaccessibility is based on the evaluation of compounds in the small bowel after upper gastrointestinal digestion, which could be performed in vivo in humans [9], in animal models [10,11], or in vitro using various digestion simulation protocols [12,13]. In vitro protocols are usually cheaper to perform [14,15], thus despite their physiological limitations, they are considered as an ideal tool for (i) early stage nutritional studies or (ii) if mechanistic investigation is required, or (iii) if the risk of toxicity of the studied material cannot be excluded. One of the most popular methods including the upper gastrointestinal phase of digestion is the Infogest protocol, which has led to a well-recognized publication of an international consensus [16]. The pH, temperature, and concentration-controlled static method simulates three main steps of human digestion: mouth, stomach, and small intestine phase. From the final small intestinal digesta (ileal fluid) of the protocol, the bioaccessibility of a wide variety of metabolites could be evaluated, such as macronutrients, minerals, and vitamins. The original method generally only uses the three major enzyme hydrolyzing macronutrients, i.e., salivary α-amylase, gastric pepsin, and pancreatin. In addition, the improved method integrated the use of gastric lipase, which is responsible for 10–25% of all lipid digestion [17]. The Infogest protocol also describes an alternative approach using individual enzymes (e.g., pancreatic lipase and trypsin) instead of enzyme complexes, such as pancreatin. It is noted, however, that if an assortment of specific purified enzymes (pancreatic lipase) is used instead of enzyme complexes, special care should be taken to include all relevant constituents, such as colipase.

Analysis of the bioaccessible fat content is a complex analytical task, since the extraction must be done from ileal fluid with a complex matrix composed of inorganic salts, bile salts, and enzymes. For the extraction of lipids from aqueous solutions (such as small intestinal digesta), the most commonly chosen method is the so-called Folch extraction, in which the solvent is a chloroform and methanol (2:1) mixture [18].

An additional difficulty in assessing the bioaccessibility of triglycerides in addition to FFAs, is partly hydrolyzed TAGs, providing MAGs also considered as bioaccessible species. These species are typically not analyzed individually in current analytical methods and simplified approaches are typically used in lipid bioaccessibility assessment studies. For instance, Fransisco et al. defined the lipid fraction remains in the aqueous phase obtained after centrifugation as bioaccessible lipids [19]. Swackhamer et al. separated the solid and liquid phase of digesta by sieving and extracted lipids of the liquid phase by solvent extraction. Finally, FA bioaccessibility was determined based on FAMEs obtained after acidic esterification [20]. Other methods apply further clean-up using SPE to remove non-polar lipids to obtain the bioaccessible FA fraction [21]. Due to the lack of consensus on one method used to isolate bioaccessible lipid fraction from digesta and the above detailed conceptual differences in approaches, in such cases, it is more relevant to refer to results as the “FFA release” obtained in the given isolated fraction. One of the most widely used routine methods in lipid digestibility studies is the pH stat method, which is based on monitoring the pH change due to liberated FAs [22]. In this work, however, a more detailed and nutritionally more relevant picture can be obtained of digestion processes if the detailed FFA profile of the digesta is determined instead of assessing total FFA quantities using, for instance, titration-based methods Thus, quantification of FFAs, while still not being fully comprehensive, nevertheless can be considered as a more informative indication of the bioaccessible FA content.

It was shown that the bioaccessibility of specific FAs could be affected by the food matrix and fatty acid position on the TAG [23,24]. Therefore, methods capable of providing FA-specific bioaccessibility information at certain extents of lipolysis are desired. Other macronutrients in the food matrix, particle size, solubility, disintegration and aggregation behavior of fat droplets, and availability for lipase absorption also affect the rate and extent of lipid hydrolysis [5]. Hence, these methods could provide valuable and relatively easy-to-perform data on FA-specific hydrolysis in studies focusing on revealing changes during co-digestion. Variation caused by the addition of various ingredients or bioactives, or the effect of different macro or microstructures (lipid droplet size, protein interaction) on the digestion of the fat content can be determined more easily via the method described in this paper.

In this paper, we describe a method suitable for the evaluation of the extent of lipolysis at the FA level using the static in vitro Infogest digestion simulation protocol (both the original and v2.0) and GC-FID technique for foods and food products. The method is specifically tailored to help routine work, which is demonstrated through the example of a baked fish meal and Orlistat, a widely applied obesity management drug.

## 2. Materials and Methods

### 2.1. Materials and Chemicals

The material for digestion protocol, rabbit gastric extract (RGE, with 15 U/mg lipase and 500 U/mg pepsin activity), was purchased from Lipolytech (Marseille, France), whereas α-amylase from porcine pancreas (>5 units/mg solid), pepsin from porcine gastric mucosa (>2500 units/mg protein), pancreatin from porcine pancreas (8× USP), porcine bile extract, as well as FAME (fatty acid methyl ester) mixture and the internals standards glyceryl nonadecanoate (C19:0 TAG; >99%), methyl nonadecanoate (C19:0 ME, analytical standard), glyceryl heptadecanoate (C17:0 TAG, >99%), heptadecanoic acid (C17:0 FFA, >99%), and Orlistat pharmaceutical secondary standard were purchased from Merck/Sigma-Aldrich (St. Louis, MO, USA). Reagents and solvents were of analytical purity. Chloroform (for analysis, stabilized with ethanol) was purchased from Carlo Erba Reagents (Val de Reuil, France), methanol (for HPLC, LC-MS grade) from VWR International (Radnor, Pennsylvania, USA), and isooctane (>95%) from Thermo Fisher Scientific (Waltham, MA, USA). High-purity water (>18 MΩ cm^−1^) was prepared by a Millipore Elix Essential 3 UV Water Purification System (Merck-Millipore (Burlington, MA, USA).

### 2.2. Sample Preparation

Common carp (*Cyprinus carpio*) from Akasztó, Hungary was provided by The Fishmarket Ltd. (Budapest, Hungary), halved and filleted. Carp fillets were cut into strips and baked at 200 °C for 20 min, to mimic realistic consumption conditions. Baked carp strips (skin and flesh) were pooled and homogenized with a Moulinex (Group SEB; Ecully, France) HV4 meat grinder, three times. This was sufficient to achieve a “mustard-like” consistency after mixing with simulated salivary solution (SSL) as suggested in the Infogest protocol [16,17]. Samples were then stored at −80 °C until measurement.

### 2.3. Methods and Equipment

The moisture content of the baked carp meal was determined according to the ISO 1442:2000 reference method [25] by oven-drying at 103 ± 1 °C using a Memmert (Schwabach, Germany) UNE300 drying cabinet. The moisture content of the digesta was also determined by oven drying at 103 ± 1 °C using the same equipment. A Heidolph (Schwabach, Germany) Laborota 4000 rotary evaporator was used to evaporate the solvent content of samples. A Hettich (Westphalia, Germany) 22R Mikro centrifuge was used for centrifugation of the samples.

### 2.4. Total Fat Content of Baked Carp

To evaluate the total fat content of the baked carp meal, the standard method available for meat and meat products was used [26]. Briefly, 5 g of accurately weighed sample portions were measured into a ceramic bowl and mixed with 15 g of acid-washed quartz sand. The mixture was dried until weight equilibrium to remove excess moisture before fat extraction. The dried sample was transferred into a paper sleeve and placed into a 50-mL Soxhlet devise. Extraction was carried out with 100 mL of petrol ether into a previously measured round-bottom flask for 6 h. After cooling, the solvent was evaporated and the weight of the dried (at 103 ± 1 °C) fat content was measured. The fat content of the sample was given in g fat/100 g food product unit.

The Bligh and Dyer fat extraction method [27]—based on Folch’s method—was tested and performed as an alternative fat extraction method. For B&D extraction, ~ 5 g of accurately weighed sample portions were measured into 50-mL centrifuge tubes with screw tops. The solvent ratio applied was calculated according to the original B&D method description, i.e., the chloroform/methanol/water ratio in the first step should be 1:2:0.8, where the moisture content of the sample gives the water ratio. Since the moisture content of the baked carp meal sample was 62.13 ± 0.17 g/100 g (determined according to [25]), in the first step, 3.9 mL of chloroform and 7.8 mL of methanol were added and vortexed for 2 min. After the addition of 3.9 mL of chloroform, the mixture was vortexed for 30 s, then 3.9 mL of water were added and vortexed again for 30 s. The two-phase solution was separated by centrifuging at 3700 *g* for 20 min. the lower phase was pipetted into a round-bottom flask with known tara weight obtained after drying until constant mass at 103 ± 1 °C, and after solvent evaporation, the extracted fat content was dried until constant mass.

FAME derivatization was performed according to [28]. Details of the protocol are summarized in the Supplementary Materials.

### 2.5. In Vitro Digestion

All digestions were made according to the Infogest protocol original version (v1.0) [16] and improved protocol (v2.0) [17]. The addition of rabbit gastric lipase is preferred in the evaluation of lipid digestion. The original method, which only applies three enzymes (amylase, pepsin, and pancreatin) was carried out and compared with the improved protocol.

All three phases were adapted and simulated stock electrolyte solutions (SSF: simulated salivary fluid, SGF: simulated gastric fluid, SIF: simulated intestinal fluid) were made as described in the consensus protocol. The enzyme activity of all used enzymes was determined via methods described in the improved digestion method [17,29]. The required volume of 6 M HCl and 1 M NaOH was previously determined during a pH test using the same amounts of samples and solvents without enzymes.

It is noteworthy to add that in v2.0, the addition of the type of RGE chosen (Lipolytech RGE15 with 15 U/mg lipase and 500 U/mg pepsin activity) contained a sufficient amount of pepsin enzyme needed to match the required activity, which omitted the use of additional pepsin enzyme in the gastric phase.

All digestion experiments were conducted with 1 g of homogenized baked carp suspended in 4 g of distilled water, in triplicates. Blank digestions were also made with each round of digestion experiment using only distilled water (5 g) to correct any lipid residue in the enzymes and bile acid added.

The residual fat content of the B&D extract of the ileal digesta after drying was investigated as follows: 5-mL aliquots of the homogenized small intestinal fluid were taken into 50-mL centrifuge tubes with screw tops and B&D extraction started with the addition of 6.25 mL of chloroform. The final B&D extract (chloroform phase) was transferred into a round-bottom flask and after solvent evaporation, the dry matter content was determined by oven drying at 103 ± 1 °C.

### 2.6. GC-FID Method

An Agilent (Santa Clara, CA, USA) 6890 GC-FID system equipped with an Agilent 7683 autosampler was used. For separation, a Phenomenex (Torrance, CA, USA) Zebron ZB-FAME (60 m, 0.25 mm, 0.20 μm) column with a cyanopropyl stationary phase and hydrogen gas (1.2 mL/min) mobile phase was used. The inlet temperature was 250 °C and the detector temperature was 260 °C. A split ratio of 50:1 and 1-µL injection volume were used. The temperature program started from 100 °C, which was kept constant for 3 min. Then, the column was heated at 20 °C/min to reach 166 °C, where it was kept for 5 min. Then, it was heated to 180 °C, at 1 °C/min, and finally to 240 °C at 10 °C/min, where it was kept for 3 min.

Calibration mix was prepared from a Supelco 37 component FAME mixture and each calibration level was spiked at 100 μg/mL with methyl nonadecanoate ISTD (1 mg/mL dissolved in isooctane). Four-point calibration was performed at a 0, 30, 60, and 120 μg/mL nominal concentration for C16: 0, at 0, 20, 40, and 80 μg/mL levels for C4:0, C6:0, C8:0, C10:0, C12:0, C14:0, C18:0, C18:1n-9c, C18:3n-6c, C18:3n-3c, C20:0, C20:3n-6c, C20:4n-6c, C20:3n-3c, and C22:0; and at 0, 10, 20, and 40 μg/mL levels for C11:0, C13:0, C14:1n-5c, C15:0, C15:1n-5c, C16:1n-7c, C:17:0, C17:1n-7c, C18:1n-9t, C18:2n-6t, C18:2n-6c, C20:1n-9c, C20:2n-6c, C21:0, C22:1n-9c, C20:5n-3c, C22:2n-6c, C23:0, C24:0, C24:1n-9c, and C22:6n-3c. In the actual calibration table, the exact concentration of each analyte was set. C4:0 was excluded from the set of analytes due to an overlap with the solvent peak. Chromatograms of the FAME mixture with internal standard and a detailed list of the analytes, trivial names, and retention times and resolution are shown in the Supplementary Materials (Figure S1 and Table S1).

### 2.7. Statistics 

Pairwise comparison using Student’s t test was carried out in Microsoft (Redmond, WA, USA) Excel. A significant difference was recognized at *p* < 0.05. Multiple comparison of release ratios of individual FAs was performed using the one-way ANOVA test in IBM (Endicott, New York, NY, USA) SPSS Statistics 25, where a significant difference was found (*p* < 0.05). Tukey’s post hoc test was performed as well. Equality of variances was tested with Levene’s test (*p* > 0.05).

### 2.8. Harmonization of the Sample Preparation Protocol

**Figure 1:**
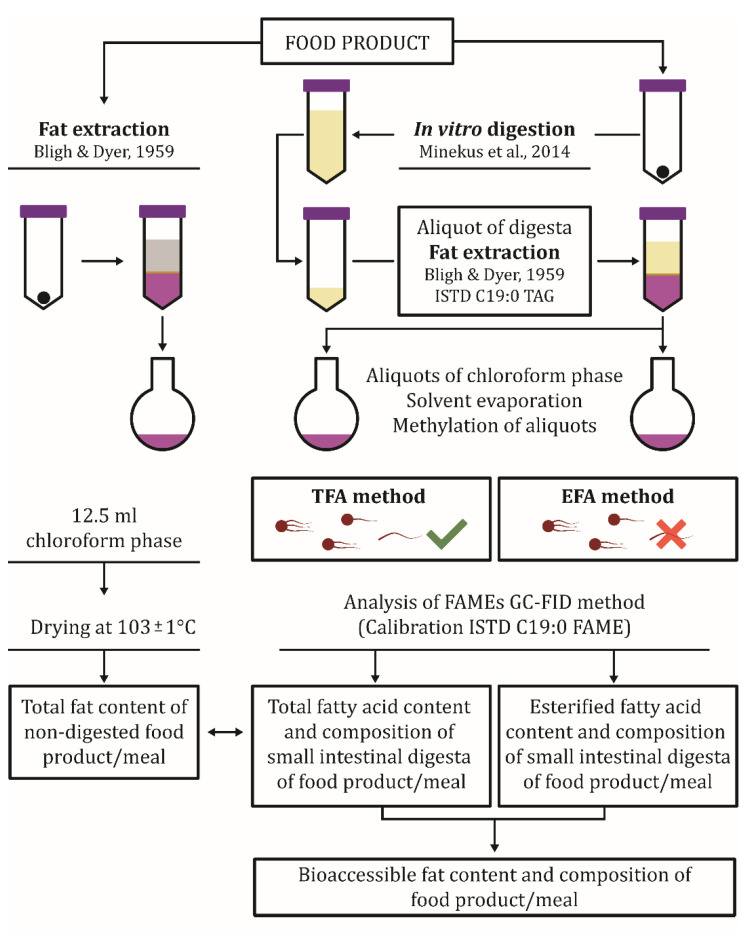
Protocol outline for harmonized sample preparation of in vitro small intestinal digest for the assessment of FFA release in food samples along with the protocol for fat content determination of the same input sample.

A harmonized protocol summarized in Figure 1 is suggested. A detailed description of the protocol including the suggested sample weight, volumes of extractants, ISTD spike concentration, and added volumes is given in the Supplementary Materials.

## 3. Results and Discussion

The desired analytical protocol aimed to be suitable for routine assessment of FFA release of different food products. Therefore, the widely used in vitro digestion protocol—the Infogest method—was chosen to simulate digestion of TAGs. Small intestinal digesta of a TAG-containing test sample simultaneously contains various fat components, such as undigested TAGs, as well as partly digested DAGs, MAGs, and FFAs. The total amount of FFAs and esterified FA species (EFA), such as DAGs, MAGs, and TAGs, in the small intestinal digesta after digestion is considered as the TFA content of the digesta [30]. The sum of released FFAs in the small intestinal lumen after digestion is used to monitor the extent of lipolysis of the ingested triacylglycerides. Quantification of lipolysis is performed by the ratio of FFAs in the final ileal fluid suspension of the digestion protocol, relative to the TFA in the same suspension. This ratio, hereafter referred to as the release ratio (RR), is considered as a measure to approximate the bioaccessibility of FAs. It is noted, however, that it is more relevant to refer to this indicator as the release ratio or FFA release instead of bioaccessibility since formed partially hydrolyzed lipids, such as MAGs and DAGs, are accounted for in the denominator of this indicator, i.e., included in TFA. The method development also included the establishment of quality control procedures, which help the routine analyst to monitor the compliance of each analytical step.

### 3.1. Fat Extraction Method

The Bligh and Dyer (B&D) method is widely accepted for the extraction of fat components from highly aqueous systems, such as small intestinal digests. Moreover, it is simple, quick, and can be used in a small scale, consuming low volumes of organic solvent. This technique was recently used for comparing the digestion and absorption rate of dietary lipid from edible oils [24] and assessing the degree of lipolysis from milk fat [31]. In this work, this protocol was the method of choice for fat extraction from the simulated small intestinal digesta.

The TFA value determined in the small intestinal digesta by the B&D method should be in agreement with the TFA content of the non-digested test food sample subjected to digestion (i.e., the input test sample). Thus, it was tested whether there was any bias in the fat content or FA profile of the digesta and the input sample due to methodological differences in the fat extraction methods. The performance of the B&D method was challenged against the ISO 1444:2000 reference method [26] for the determination of the fat content and fatty acid profile of the baked carp meal, the selected specimen food.

The fat content of the product obtained from the above B&D protocol was compared to the ISO 1444:2000 standard method (applying Soxhlet extraction with petroleum ether) designed for evaluating the fat content of meat and meat products. Both B&D and ISO reference fat extractions were carried out in triplicates and results were compared using Student’s t test. The fat content measured by the B&D method (14.44 ± 0.30 g/100 g baked carp meal) is considered equivalent to the reference method (14.05 ± 0.78 g/100 g baked carp meal, *t* test: *p* = 0.292), thus it is considered suitable for determination of the fat content of the tested food product. In addition to total fat comparison, FA profiling (using the TFA method) of the extracted fat of the baked carp meal was also performed. No significant differences were found (*p* = 0.001) for any of the FAs at >1% contribution to TFA (data not shown).

As a proposed quality assurance method, the assessment of TFA content (FFAs and EFAs) in the B&D extract of the small intestinal digesta by weight was also considered and the applicability of this approach was tested. First, the moisture content of digesta was determined (96.27 ± 0.003 g/100 g; *n* = 6) to assess the required solvent volumes. Determination of the residual fat content of the B&D extract of the ileal digesta after drying was performed. It was found that the obtained dry matter in the B&D extract of the small intestinal digesta (6.14 ± 0.65 g/100 g baked carp meal) was significantly less than the fat content determined in the input sample either by B&D or the reference fat extraction method (*p* < 0.001 for t tests in the comparison to both fat extraction methods). The weight loss during drying is most probably caused by the evaporation or degradation (such as oxidation) of FFAs in the extracted fat portion of the digesta. Since the extent of lipid digestion is above 50% (i.e., most TAGs are in the form of FFAs in the digested samples), it explains the high loss due to the presence of FFAs. Conclusively, this approach, which includes drying of the digested sample, is not suitable and should not be used to assess the TFA content in the B&D extract of the small intestinal digesta.

Finally, the FA profiles of the fat extracted by the ISO 1444:2000 standard and the fat extracted from the small intestinal digesta by the B&D method were compared. No significant differences were found (*p* = 0.001) for any of the FAs at >1% contribution to TFA (data not shown). Conclusively, the TFA content of the non-digested input sample is equivalent to the TFA content determined in the small intestinal digesta after B&D extraction, thus the TFA content of the digesta can be used as a reference quantity for calculations.

### 3.2. Determination of Fatty Acid Release

Fatty acids are usually determined by gas chromatography after fat extraction and derivatization of fatty acid methyl esters. Several methods are available in the literature depending on the studied food product, evaluated components, in-house routine, and availability of different standard methods. Since the majority of FAs are in esters in most food commodities, FA profiling in food analysis almost exclusively focuses on the analysis of esterified forms of FAs and the FFA fraction is often neglected. Thus, derivatization is most often carried out by alkaline transmethylation, i.e., FAMEs are directly methylated from triacylglycerides, whereas soaps formed from FFAs are ignored. On the other hand, the selective determination of FFAs, which is crucial in bioaccessibility assessment, is a more complex task. One of the options for analyzing FAMEs derived from FFAs is the boron-trifluoride method. Although this is a standard technique, the method uses highly poisonous reagents, and most laboratories prefer to avoid it. Thus, it was not considered as an option in the proposed routine protocol. As an alternative approach, the amount of an individual FFA (FFA_i_) can be determined as the difference between the total fatty acid value of the given FA (TFA_i_) and the esterified fatty acid value of the same FA (EFA_i_), as suggested by [30] and given in Equation (1).

In this work, this approach was chosen, and derivatization protocols were based on the ones described in the EN ISO 12966-2:2017 standard [28]. Briefly, determination of TFAs relies on a two-step reaction, in which the first alkaline reaction transesterifies acylglycerides into FAMEs and saponifies FFAs. The formed soaps are methylated in the second acid-catalyzed methylation step. This method, hereafter referred to as the *TFA* method, converts all types of fatty acid components (tri-, di-, monoacylglycerides and FFAs) in methylated form, independently of being esterified or free. It should be noted that with this approach, soaps formed during digestion (e.g., insoluble Ca soaps of saturated long-chain FAs [32]) are also accounted for as part of the TFA pool, thus apparently increasing the bioaccessible FA content.

The EFA fraction is determined by applying only the alkaline transmethylation derivatization step, in which only the esterified acylglycerides transform into FAMEs, whereas FFAs are saponified. This method is hereafter referred to as the *EFA* method. Based on the analytical results obtained by the two approaches described above, the release ratio (RR) of any individual FFAs was calculated as the amount (in g/100 g sample portion subjected for digestion) of an individual FFA (FFA_i_) in the small intestinal digesta, divided by the amount (also in g/100 g sample) of TFA obtained for the same FA (TFA_i_) in the small intestinal digesta, as given in Equation (2). Similarly, the total amount of released FA content (in %) can be calculated as the sum of FFAs divided by the sum of TFAs, as given in Equation (3):

FFA_i_ = TFA_i_ − EFA_i_ (1)

RR_i_ = FFA_i_/TFA_i_ (2)

Total released FA content [%] = (∑FFA_i_/∑TFA_i_) × 100 (3)

where TFA_i_ is the total (free and esterified) amount of an individual FA in the small intestinal digesta (expressed as g/100 g sample), EFA_i_ is the amount of the same FA in esterified (undigested) form, FFA_i_ is the amount of the same individual free FA in the small intestinal digesta, ∑FFA_i_ is the sum of the amount of each individual FFAs, and ∑TFA_i_ is the sum of the amount of each individual FA in the small intestinal digesta.

It should be noted that the amount of FFAs measured in the small intestinal digesta also implicitly includes the FFAs already available in the sample prior to digestion and FAs formed during digestion from other types of lipid molecules, such as phospholipids. From a nutritional point of view, however, it is relevant, since FFAs already in the sample before digestion, either naturally or added as a food additive (e.g., E570), are metabolized in the same way as FFAs liberated from TAGs during digestion [33]. Nevertheless, if necessary, the FFAs of the input test food/meal can also be determined with our proposed method using the same extraction and methylation procedures followed by the same GC-FID method.

### 3.3. Internal Standardization

The analytical methods used for evaluating FA composition usually use one or two internal standards (ISTD) to cover the full range of analytes. The ISTDs are typically added after fat extraction and methylation in the form of methyl esters. The primary purpose of the ISTD used in this way is to correct for any differences in sensitivity experienced during calibration and sample measurement. However, this technique does not correct/compensate for the incidental sample losses, which could occur during the extensive multistep sample preparation operations. Even the standard methylation protocol [28] draws attention for the possible occurrence of sample losses during heating and sample transfer, which we aimed address in our proposed method. Therefore, it was necessary to find an internal standard, which can be added as early as the first fat extraction step and is suitable for monitoring the compliance of the entire sample preparation process and for correcting any occurring errors. In addition, the proposed ISTD should be amenable to fit the FA profiling protocol applied in the case of the non-digested (input) samples as well as in the sample preparation protocols used to assess the released FFA fraction of the small intestinal digesta.

Fatty acid methyl esters are commonly used ISTDs [24]. These molecules are advantageous, since they are already in an appropriate form for GC analysis, and are extractable with the B&D method. However, these compounds cannot correct for any errors that occur during methylation. As an alternative, an appropriate FFA—that is not available endogenously in the sample—could be considered; however, FFAs as ISTDs cannot simulate and thus correct for any errors that may occur during the hydrolysis step. Eventually, we decided to use triacylglycerides as ISTDs. TAGs can be co-extracted in the extraction step and react in the methylation process used to evaluate the released FA content in the protocol. Glyceryl trinonadecanoate was selected, since C19:0 ME elutes in the middle range of the chromatogram of the studied FAs, thus representing both short-, middle-, and long-chain fatty acids and does not occur naturally or as an additive in any foodstuff or food product [34].

### 3.4. Recovery

Two test compounds, namely glyceryl triheptadecanoate and heptadecanoic acid, were chosen for the recovery study. C17:0 TAG represents the esterified non-digested FAs, whereas C17:0 FFA represents free fatty acids. This combined use of a TAG and a FFA for a recovery study was chosen since it can comprehensively validate the suitability of the proposed method to accurately determine both EFA and FFA species in the small intestinal digesta. It is crucial, since FFAs and the sum of FFAs plus EFAs (i.e., TFAs) are used to quantitate RR. This experiment can also implicitly validate the appropriateness of B&D extraction for both FFAs and EFAs from digesta, as well as the use of C19:0 TAG as an internal standard.

In this experiment, a blank digest using 5 g of water as a sample and baked carp digest using 1 g of homogenized baked carp meal suspended in 4 g of distilled water were prepared using the Infogest in vitro digestion protocol. From each digest, 1-mL homogenized aliquots were transferred to 50-mL centrifuge tubes and 250 μL of 1 mg/mL C19:0 TAG (dissolved in chloroform, CHCl_3_) were added as ISTD. Half of the samples were also spiked with 250 µL of 1 mg/mL C17:0 TAG (in CHCl_3_) and the other half with 250 µL of 1 mg/mL C17:0 FFA (in CHCl_3_) before performing the B&D extraction.

FA profiling of the digesta was carried out by the *TFA* method, resulting in FAMEs of all the tri-, di-, and monoacylglycerols and FFAs of the sample, whereas the *EFA* method included only FAMEs originating from esterified triglycerides. It was postulated that the spiked amount of C17:0 TAG would be recovered with both methods; however, spiked C17:0 FFA would only be recovered with the *TFA* method.

To extract fat components with the B&D method, 0.75 mL of pure chloroform and an additional 0.5 mL of chloroform as a vehicle of spiked C19:0 TAG and C17:0 compounds were added to the digesta samples. Next, 2.5 mL of methanol were also added, and samples were stirred vigorously for 2 min. After addition of the next 1.25 mL of chloroform portions, samples were vortexed for 30 s, then another 1.25 mL of water were added, and samples were vortexed for 30 s again. Finally, tubes were centrifuged for 20 min at 3700 *g*. From the bottom (chloroform) layer, 1-mL aliquots (containing 100 ug/mL of C17:0 FFA or TAG) were taken into 50-mL round-bottom flasks. After solvent evaporation, derivatization according to the *TFA* and *EFA* methods was carried out as described in the protocol. The results of the recovery experiment are shown in Table 1.

As presumed in samples spiked with C17:0 TAG, both methylation methods transformed test components into their FAME forms. The recoveries of C17:0 TAG varied in the range of 105–108%. The pairwise comparison of C17:0 ME’s concentration [µg/mL] from the *TFA* and *EFA* methods showed no significant differences in the C17:0 ME, neither in the blank (*p* = 0.127) nor in the baked carp meal digesta (*p* = 0.526). However, in samples spiked only with C17:0 FFA, C17:0 ME could only be detected with the *TFA* method. The recovery of spiked C17:0 FFA methylated with the *TFA* method was in the range of 104–106%. Comparing the results of the blank and the matrix samples, the C17:0 ME concentrations obtained by the *TFA* method showed no significant differences (blank: *p* = 0.550; baked carp: *p* = 0.912). On the contrary, no C17:0 ME signals could be detected by the *EFA* method since no detectable amount of endogenous C17:0 TAG was found in the carp and the *EFA* method did not transform spiked C17:0 FFA to its methyl esters. Our results show that the proposed method applying the B&D extraction and C19:0 TAG as an ISTD is suitable for accurate determination of both FFAs and EFAs.

### 3.5. Application to Baked Carp Meal

First, the TFA composition of the baked carp meal test matrix was evaluated from baked carp digesta. Relevant FAs (above 1 *w*/*w*% of carp meal) in this sample were oleic acid (C18:1n-9c: 52.8 ± 0.3%), palmitic acid (C16:0, 18.4 ± 0.2%), linoleic acid (C18:2n-6c, 9.0 ± 0.1%), palmitoleic acid (C16:1n-7c; 7.1 ± 0.0%), stearic acid (C18:0, 5.9 ± 0.1%), gondoic acid (C20:1n-9c: 2.7 ± 0.1%), and α-linolenic acid (C18:3n-3c, 1.1 ± 0.1%). The sum of these FAs gives 97.0% of all FAs detected in the sample. The detailed FA composition is presented in Table 2.

The released FA content was measured via the two versions of the Infogest consensus protocol. Infogest v1.0 uses only pancreatin whilst v2.0 highlights the importance of gastric lipid digestion and adds rabbit gastric lipase in the stomach phase of the method.

**Figure 2:**
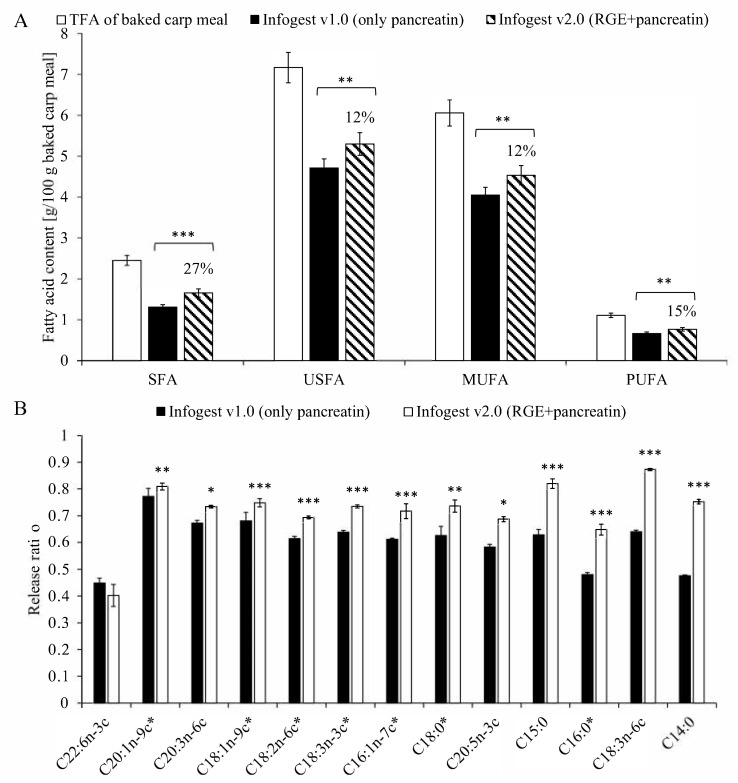
(**A**): Saturated fatty acid content (SFA), unsaturated fatty acid content (USFA), monounsaturated fatty acid content (MUFA), and polyunsaturated fatty acid content (PUFA) of a baked carp meal expressed as g/100 g baked carp meal. Total fatty acid content (TFA) was determined from small intestinal digesta via the *TFA* method (blank column), and the released fatty acid content after Infogest v1.0 (black column) and Infogest v2.0 (hatched column) was calculated as given in Equation (1). The difference between the released FA composition via v1.0 and v2.0 is given in % above the respective columns. (**B**): Release ratio (RR) of individual fatty acids in the baked carp meal (Equation (2)). Asterisks following FA abbreviations show FAs occurring at >1 w/w% in the sample. Comparison between Infogest v1.0 (black column) and v2.0 (blank column). Significance determined via the t test shown: *: *p* < 0.05; **: *p* < 0.01; ***: *p* < 0.001. RGE: rabbit gastric lipase.

The proposed method is suitable for providing FA-specific RR data. It can be concluded that independent of the applied v1.0 or v2.0 protocol, FA-specific significant differences (*p* < 0.0001) can be seen in the RR of different FAs (Figure 2). In the studied sample type, RR values varied between 0.45 and 0.77 for the v1.0 protocol and 0.40 and 0.87 for v2.0. It is noteworthy that the RR value of C16:0, the second most abundant FA in the baked carp meal, is remarkably below the total released FA content in both cases.

The total released FA content (calculated according to Equation (3)) was 62.8 ± 1.5% and 72.3 ± 0.9% for the v1.0 and v2.0 protocols, respectively. This observed 9.5% increase in the total released FA content correlates with data from the literature on the effect of gastric lipase, i.e., adds 10–25% to all-over lipolysis [16]. Based on the results obtained from the comparison of protocol v1.0 and 2.0, the contribution of gastric lipase can be characterized. The FA-specific analysis of the results shows that gastric lipase has a biased relative contribution to the liberation of FAs. Its contribution to the release of saturated FAs was more than double (+27%) that of the unsaturated species (+12%) (see Figure 2A). Our FA-specific comparison shows that the most significant difference in the RR was for C14:0. This has a minor role in the increase in total SFA released but may be of nutritional significance [2,35]. Conclusively, the increase in the RR, primarily of C16:0 and to lesser extent C18.0, is most probably responsible for the majority of the observed change in the total amount of released SFAs due to their dominancy amongst FAs. A further important role of gastric lipase in relation to C16:0 can be postulated from our observations. The RR of C16:0 was among the lowest (RR = 0.48) ones without gastric lipase, which showed one of the largest increases (~35%) attributable to RGE (RR = 0.65). Moreover, it is worth mentioning that the observed RR change of C18:1 (oleic acid) caused by the addition of RGE is below the overall average increase; however, being the most abundant FA in the baked carp meal, its contribution to the total released FA pool is of importance (see Figure 2B).

Our results obtained from the studied baked carp meal clearly demonstrate that assessment of lipolysis at the level of individual FAs has importance in nutritional studies, e.g., the health benefits and risks of SFAs and mono- or polyunsaturated FAs (MUFAs and PUFAs) should be evaluated separately. The results distinctively show that the FA profile of non-digested food products and the released FA content and composition of the digests provided by the two versions of the Infogest consensus protocol can be significantly different. Individual FAs occurring in a food or food product might show different RRs, which could be related to matrix effects, TAG composition, or the specific substrate preference of pancreatic or gastric lipase toward different FAs [36].

### 3.6. Indication of Lipase Inhibition with Orlistat

Orlistat is currently the only clinically approved drug for obesity management in Europe. The molecule inhibits pancreatic lipase activity, thus helping in the reduction of triacylglyceride bioaccessibility. This substance was chosen in this study as a positive validation control to demonstrate the capability of our proposed method to indicate shifts in the bioaccessibility of TAGs in a real food sample as a result of treatment with a compound with proven lipase inhibition potential. For this purpose, in vitro digestion of the baked carp meal amended with Orlistat and determination of the released FA content was carried out as the proposed protocol. The addition of 40 µL of 0.5 M Orlistat solution (in DMSO), resulting in a 0.5 mM concentration in the final small intestinal digesta, was performed at the beginning of the oral stage of the digestion protocol. The concentration of Orlistat solution added was chosen between the amount in the approved drug (Xenical) and the amount appropriate to stop all lipid activity as seen in [37].

Our results show a remarkable decrease in the released FA content of the Orlistat-treated baked carp sample. The total released FA content was only 2.1 ± 0.9%. This result was further cross validated by determining the fat content (by weight) in the B&D chloroform extract of the digesta after evaporation and drying at 103 ± 1 °C. It was found that the fat content in the extract was not significantly different from the sum of FAs measured by the *TFA* method (*t* test, *p* = 0.239). This result also indirectly lends support to the previously described observation that when a high percentage of FFAs form during digestion, which tend to evaporate or degrade more easily during evaporation and drying, the fat content measured by weight in the digested small intestinal fluid is not in agreement with the sum of FAs measured by the *TFA* method.

## 4. Conclusions

Although evaluation of the fat content and composition of food products is a routine task, measurement of FA-specific lipolysis of the triacylglyceride content in intestinal digesta of real food samples is still a challenge. The intestinal digesta as a special matrix and the need for determining both FFAs and esterified FAs simultaneously requires customized sample preparation and internal standardization protocols. The method described in this paper describes a comprehensive, nevertheless easy-to-follow, protocol for the robust determination of lipolysis at the FA level from small intestinal digesta, resulting in the standardized in vitro Infogest protocol.

The developed harmonized analytical protocol (extraction, internal standardization, dilutions) is designed in a way to result in uniform samples from the point of view of the GC-FID fatty acid profiling method. Either undigested input (food) samples or small intestinal digesta samples can be subjected to the same generalized EFA or TFA derivatization protocols and the resulting analytical samples are conveniently amenable for GC-FID analysis using a generalized calibration for a wide variety of FAMEs.

Advantages of the protocol include (i) its tailoring to the widely accepted Infogest digestion protocol; (ii) derivatization of FAMEs made with established methods published in ISO 12966-2:2017 standard, without using highly poisonous reagents or excess amounts of organic solvents; (iii) correction of minor sample losses during preparation using unique internal standardization with commercially available ISTDs; (iv) both total FA content and composition and FFA content and composition of food products can be conveniently analyzed by following the protocol; (v) released FAs in food products can be approximated either as the total released FA content (in %) or at the individual FA level (RR); and (vi) our method is also an appropriate choice for lipase inhibition studies with real food matrices. The latter is of great importance since in the last few years, many efforts have been made to gain evidence regarding the lipase inhibition potential of several bioactive compounds [38]. This method could contribute to broadening the knowledge in this field by providing more detailed FA-specific information on the characteristics of bioaccessibility. A limitation of the proposed method is that is assesses TAG bioaccessibility by focusing on released FAs and does not consider MAGs. However, unlike methods approximating fat bioaccessibility without dedicated selectivity to either of MAGs or FFAs (the two major lipolytic product of TAG digestion) [19,20,21], with this method, released FFAs can be selectively determined. Current results on the similarity/dissimilarity in the uptake and transfer properties of these two classes of lipolytic digestion products of TAGs are still controversial [39], thus distinguishing between MAGs and FFAs among bioaccessible species might be of importance in further studies. Our proposed method is a suitable candidate for such purposes.

## Figures and Tables

**Table 1:** Results of the recovery experiment. Concentration of heptadecanoic acid methyl ester [µg/mL] in samples spiked with same amount of C17:0 TAG or C17:0 FFA, derivatized with ISO 12966-2:2017 standard method’s *TFA* method and *EFA* method as detailed in the text. The C19:0 TAG was spiked at 100 µg/mL to all samples as well as in calibration solutions as ISTD.

Added Standard	C17:0 TAG		C17:0 FFA	
Derivatization Method	*TFA* Method	*EFA* Method	*TFA* Method	*EFA* Method
Blank Digest	108.38 ± 1.96	106.06 ± 0.71	104.25 ± 10.79	n/a
Baked Carp Digest	105.01 ± 6.09	107.99 ± 4.25	105.56 ± 5.32	n/a

C17:0 TAG: glyceryl triheptadecanoate; C17:0FFA: heptadecanoic acid; *TFA* and *EFA method*: Total Fatty Acid and Esterified Fatty acid method, respectively, as detailed in the text; n/a: not applicable.

**Table 2:** Fatty acid composition of the baked carp meal’s small intestinal digesta: total fatty acid content and free fatty acid content in mg/100 g baked carp meal (calculated according to Equation (1)) via Infogest v1.0 and v2.0. Contribution of the given FA in % is shown in parentheses. Release ratio (RR) of individual fatty acids was calculated according to Equation (2) via Infogest v1.0 and v2.0 (results are presented in bold).

FAs		Total Fatty Acid Content *		Free Fatty Acid Content (Infogest v1.0)		RR (Infogest v1.0)	Free Fatty Acid Content (Infogest v2.0)		RR (Infogest v2.0)
#	Abbr.	mg/100 g Baked Carp Meal(%)	RSD	mg/100 g Baked Carp Meal(%)	RSD		mg/100 g Baked Carp Meal(%)	RSD	
-	C4:0	n/a	-	n/a	-	-	-	n/a	-
1	C6:0	n/a	-	n/a	-	-	-	n/a	-
2	C8:0	n/a	-	n/a	-	-	-	n/a	-
3	C10:0	n/a	-	n/a	-	-	-	n/a	-
4	C11:0	n/a	-	n/a	-	-	-	n/a	-
5	C12:0	n/a	-	n/a	-	-	-	n/a	-
6	C13:0	n/a	-	n/a	-	-	-	n/a	-
7	C14:0	77.8 ± 3.7 (0.8)	0.05	40.4 ± 1.1 (0.7)	0.03	**0.48 ± 0.06**	58.5 ± 3.5 (0.8)	0.06	**0.75 ± 0.03**
8	C14:1n-5c	n/a	-	n/a	-	-	-	n/a	-
9	C15:0	10.2 ± 0.5 (0.1)	0.05	7 ± 0.4 (0.1)	0.05	**0.63 ± 0.07**	8.7 ± 1.1 (0.1)	0.13	**0.85 ± 0.08**
10	C15:1n-5c	n/a	-	n/a	-	-	-	n/a	-
11	C16:0	1768.8 ± 86.4 (18.4)	0.05	867.7 ± 36.8 (14.5)	0.04	**0.48 ± 0.02**	1145.8 ± 55.1 (16.5)	0.05	**0.65 ± 0.01**
12	C16:1n-7c	685.8 ± 34.4 (7.1)	0.05	412.3 ± 15 (6.9)	0.04	**0.61 ± 0.02**	491.6 ± 24.3 (7.1)	0.05	**0.72 ± 0.01**
13	C17:0	11 ± 0.8 (0.1)	0.07	7.5 ± 0.6 (0.1)	0.07	**0.63 ± 0.09**	8.2 ± 1.1 (0.1)	0.13	**0.75 ± 0.07**
14	C17:1n-7c	n/a	-	n/a	-	-	-	n/a	-
15	C18:0	566.3 ± 30.4 (5.9)	0.05	362.8 ± 25.5 (6)	0.07	**0.63 ± 0.01**	417.4 ± 44.9 (6)	0.11	**0.74 ± 0.06**
16	C18:1n-9t	n/a	-	n/a	-	-	-	n/a	-
17	C18:1n-9c	5079 ± 266.6 (52.8)	0.05	3415.2 ± 160 (56.9)	0.05	**0.68 ± 0.01**	3799.9 ± 194.1 (54.6)	0.05	**0.75 ± 0.01**
18	C18:2n-6t	n/a	-	n/a	-	-	-	n/a	-
19	C18:2n-6c	866.5 ± 45 (9)	0.05	525 ± 23.2 (8.7)	0.04	**0.61 ± 0.01**	600.7 ± 28.3 (8.6)	0.05	**0.69 ± 0.01**
20	C18:3n-6c	11.4 ± 0.6 (0.1)	0.05	7.3 ± 0.9 (0.1)	0.13	**0.64 ± 0.04**	10.2 ± 1.1 (0.1)	0.11	**0.89 ± 0.06**
21	C18:3n-3c	102.2 ± 5.9 (1.1)	0.06	65.2 ± 4.6 (1.1)	0.07	**0.64 ± 0.02**	75.1 ± 5 (1.1)	0.07	**0.73 ± 0.02**
22	C20:0	n/a	-	n/a	-	-	-	n/a	-
23	C20:1n-9c	262.4 ± 16.5 (2.7)	0.06	206.1 ± 15 (3.4)	0.07	**0.77 ± 0.01**	212.5 ± 17 (3.1)	0.08	**0.81 ± 0.02**
24	C20:2n-6c	n/a	-	n/a	-	-	-	n/a	-
25	C21:0	n/a	-	n/a	-	-	-	n/a	-
26	C20:3n-6c	36.2 ± 1.9 (0.4)	0.05	25.3 ± 2.2 (0.4)	0.09	**0.67 ± 0.02**	26.6 ± 2.3 (0.4)	0.09	**0.73 ± 0.04**
27	C20:4n-6c	n/a	-	n/a	-	-	-	n/a	-
28	C20:3n-3c	n/a	-	n/a	-	-	-	n/a	-
29	C22:0	n/a	-	n/a	-	-	-	n/a	-
30	C22:1n-9c	7.4 ± 0.6 (0.1)	0.08	6.9 ± 0.7 (0.1)	0.10	**0.91 ± 0.08**	5.7 ± 2.5 (0.1)	0.44	**0.76 ± 0.33**
31	C20:5n-3c	42.8 ± 2.2 (0.4)	0.05	25.7 ± 1.7 (0.3)	0.11	**0.58 ± 0.06**	31.7 ± 6.5 (0.5)	0.20	**0.74 ± 0.13**
32	C22:2n-6c	n/a	-	n/a	-	-	-	n/a	-
33	C23:0	7.9 ± 1.5 (0.1)	0.19	6.4 ± 1.6 (0.1)	0.25	**0.74 ± 0.19**	6.8 ± 1 (0.1)	0.15	**0.87 ± 0.1**
34	C24:0	n/a	-	n/a	-	-	-	n/a	-
35	C24:1n-9c	n/a	-	n/a	-	-	-	n/a	-
36	C22:6n-3c	47.5 ± 2.8 (0.5)	0.06	24.1 ± 3.7 (0.4)	0.15	**0.45 ± 0.04**	19.2 ± 4.5 (0.3)	0.23	**0.4 ± 0.08**

n/a: not applicable; results given in average± SD (*n* = 6); RSD: relative standard deviation; * TFA content shown here was calculated from digesta obtained via v2.0. Since there is no significant difference between TFA of v1.0 and v2.0, data of v1.0 is not shown here.

## Data Availability

MDPI Research Data Policies at https://www.mdpi.com/ethics (accesed on 23 September 2021).

## Supplementary Materials

The following are available online at https://www.mdpi.com/article/10.3390/nu13113889/s1, Figure S1: Chromatograms of (A) FAME mixture (calibration level 4, spiked with 100 µg/mL C19ME), (B) small intestinal digesta of baked carp meal: (1) *TFA* method, (2) *EFA* method. (see text for details) Numbers on Figure correspond to peak numbers shown in Table 2. Table S1: Detailed list of analytes, including abbreviations, compound names, trivial names, retention times (R_t_) [min] and resolution (R) [-].
