# Supplementary material for: Assessment of Fatty Acid-Specific Lipolysis by In Vitro Digestion and GC-FID
Source: Nutrients. 2021 Oct 29;13(11):3889. doi: 10.3390/nu13113889 (PMC8623358; doi:10.3390/nu13113889)
Supplement: Supplementary file 1 [file nutrients-13-03889-s001.zip › nutrients-1413537-supplementary.pdf]

nutrients

Supplementary Materials: Assessment of Fatty Acid-Specific Lipolysis by In Vitro Digestion and GC-FID

Judit Tormási and László Abrankó *

1. Harmonized Protocol for the Determination of FA Release after In Vitro Infogest Digestion Simulation

1.1. Evaluation of the Fat Content of the Input Food Product

Five grams of accurately weighed sample portions (in the form to be subjected for digestion) were measured into 50-mL centrifuge tubes with screw tops. The required volumes of extractants are moisture content dependent, thus the moisture content of the sample should be previously measured. The chloroform/methanol/water ratio in the first step should be 1:2:0.8, where the moisture content of the sample gives the water ratio. Thus, in the first step, if the moisture content of the test sample was, for instance, 60%, 3.75 mL of chloroform and 7.5 mL of methanol were added, and the suspension was vortexed for 2 min. Next, the sample was amended with the same volume of chloroform as in the first step and vortexed for 30 s. Then, the same volume of distilled water was added and vortexed again for 30 s. Two-phase solution was separated via centrifugation at 3700 *g* for 20 min. The lower chloroform phase was pipetted into a round-bottom flask with a known tara weight obtained after drying until constant mass at 103 ± 1 °C. Solvent was then evaporated with a rotary evaporator, then the flask was oven dried at 103 ± 1 °C until constant mass. The fat content was calculated from the net dry mass of the extract divided by the weight of the test portion.

1.2. In Vitro digestion

In vitro digestion was carried out according to the IFOGEST protocols as given in Minekus et al., 2014 [16] or Brodkorb et al., 2019 [17]. Briefly, 5 g of sample were accurately measured into a pre-weighed 50-mL centrifuge tube. For the oral phase, 3.5 mL of SSF (tempered to 37 °C), 25 µL of 0.3 M $CaCl_2$, 0.5 mL of amylase solution (1500 U/mL in SSF), and 0.975 mL of distilled water were added. Homogenized samples were incubated in an overhead shaker (Heidolph Reax 2) fitted inside a preheated drying cabinet (Memmert UNE300) for 2 min at 37 °C. In the gastric phase, 6.4 mL of SGF (tempered to 37 °C), 5 µL of 0.3 M $CaCl_2$, 50 µL of 6 M HCl, 1.6 mL of pepsin solution (25,000 U/mL in SGF), and 1.945 mL of water were added, and the mixture was incubated in the overhead shaker at 37 °C for 2 h. For the small intestine phase, 8.5 mL of SIF (tempered to 37 °C), 40 µL of 0.3 M $CaCl_2$, 2.5 mL of bile extract solution (160 mM in SIF, tempered to 37 °C), 5 mL of pancreatin solution (800 U/mL in SIF), 100 µL of 1 M NaOH, and 3.86 mL of water were added, and the mixture was incubated in the overhead shaker at 37 °C for another 2 h. In the case of the Infogest v2.0 digestion [17], 1.6 mL of rabbit gastric extract and RGE (750 U/mL in SGF) were added in the gastric phase and no pepsin was added. After the small intestinal digestion phase was completed, the weight of the digests was measured. Aliquots of 0.5 mL were immediately taken from each digest and transferred to pre-weighed 50-mL tubes. The weight of the aliquot was recorded for later calculations. The sample was diluted with 4.5 mL of water, and 6 mL of chloroform intended for fat extraction were immediately added to quench enzymatic digestion processes.

1.3. Fat Extraction

Before fat extraction, 250 µL of C19:0 TAG internal standard (ISTD) solution (1 mg/mL in $CHCl_3$) were added to the samples. Then, 12.5 mL of methanol were added, and the sample was vortexed for 2 min using a Benchmark Benchmixer XL. Then, another 6.25 mL of chloroform were added, followed by 30 s of vortexing. Finally, after the addition of

6.25 mL of distilled water, the sample was vortexed again for 30 s. The formed two-phase solution was centrifuged at 3700 *g* for 20 min.

1.4. FAME Derivatization

After phase separation, from the lower phase (∑12.5 mL of chloroform, containing fat components), 2 × 5 mL was pipetted into two round-bottom flasks and solvent was evaporated using a rotary evaporator. One aliquot was methylated according to the ISO 12966-2:2017 standard's '*General method'* to obtain the total fatty acid content (*TFA method*) while the other aliquot was transmethylated with the alkaline '*Fast method'* of the same ISO standard to obtain the esterified fatty acid content (*EFA method*).

1.4.1. Determination of the Total Fatty Acid Content (TFA Method)

After evaporation of 5 mL of chloroform, samples were methylated in the same round-bottom flask. First, chips were boiled and 2 mL of 0.2 M sodium methoxide were added, mixed, and heated for 20 min in a sand bath. Cooled samples were titrated in the presence of phenolphthalein using 1 M sulfuric acid dissolved in methanol, in 100-µL portions until discoloration. Then, another 200 µL of acid were added in excess. Acidified samples were heated for another 5 min, then removed from the heat and cooled under running water. Then, the sample was transferred into a 15-mL screw-top centrifuge tube, and the flask was washed with 4 mL of saturated (40 g/100 mL) sodium chloride solution. The sample was homogenized before extraction of formed fatty acid methyl esters (FAMEs) by the addition of 1 mL of isooctane. Two-phase solution was separated by centrifugation at 3700 *g* for 10 min before transferring the upper phase into a GC vial.

Note: Sodium methoxide solution must be added right before heating, since the hydrolysis reaction requires heat. If the alkaline solution is added without heating, the reaction will be less effective, resulting in a poor yield.

1.4.2. Determination of the Esterified Fatty Acid Content (EFA Method)

The total amount of dried fat extract in the bottom of the round-bottom flask was dissolved in 1 mL of isooctane. The dissolved sample was completely removed (pipetted) into a 15-mL screw-top centrifuge tube. To methylate the esterified components, 100 µL of 2 M potassium hydroxide (dissolved in methanol) were added and vigorously vortexed for 1 min. After the reaction, the sample became opaque, which cleared after 2 min of resting. Then, 4 mL of saturated (40 g/100 mL) sodium chloride solution were added and vortexed. Two-phase solution was centrifuged at 3700 *g* for 10 min before transferring the upper phase to another vial already containing 0.5 g of Na_2SO_4 powder to remove excess moisture from the samples. Finally, an aliquot of the sample was pipetted into a GC vial for analysis. Samples were analyzed with the GC-FID method described in the Experimental methods chapter of the main text.

Table S1. Detailed list of analytes, including abbreviations, compound names, trivial names, retention times (R_t) [min], and resolution (R) [-].

Abbr.	Compound	Trivial Name	R_t [min]	R [-]
C4:0	Methyl butanoate	Butyric acid	-	-
C6:0	Methyl hexanoate	Caproic acid	4.32	-
C8:0	Methyl octanoate	Caprylic acid	5.44	58.9
C10:0	Methyl decanoate	Capric acid	6.78	72.4
C11:0	Methyl undecanoate	Undecylic acid	7.49	38.4
C12:0	Methyl laurate	Lauric acid	8.22	37.7
C13:0	Methyl tridecanoate	Tridecylic acid	9.02	37.9
C14:0	Methyl myristate	Myristic acid	9.94	39.0
C14:1n-5c	(Cis-9) Methyl myristoleate	Myristoleic acid	10.63	26.0

C15:0	Methyl pentadecanoate	Pentadecylic acid	11.05	14.4
C15:1n-5c	(Cis-10) Methyl pentadecenoate	Pentadecenoic Acid	11.89	27.0
C16:0	Methyl palmitate	Palmitic acid	12.43	15.8
C16:1n-7c	(Cis-9) Methyl palmitoleate	Palmitoleic acid	13.24	21.8
C17:0	Methyl heptadecanoate	Margaric acid	14.16	22.6
C17:1n-7c	(Cis-10) Methyl heptadecenoate	Heptadecenoic acid	15.12	22.1
C18:0	Methyl stearate	Strearic acid	16.30	24.8
C18:1n-9t	(Trans-9) Methyl octadecenoate	Elaidic acid	16.89	11.7
C18:1n-9c	(Cis-9) Methyl oleate	Oleic acid	17.20	6.0
C18:2n-6t	(all Trans-9,12) Methyl linoleaidate	Linolelaidic acid	18.20	18.4
C19:0 (ISTD)	Methyl nonadecanoate	Nonadecylic acid	18.89	12.1
C18:2n-6c	(all-Cis-9,12) Methyl linoleate	Linoleic acid	19.04	2.6
C18:3n-6c	(all-Cis-6,9,12) Methyl linolenate	γ-linolenic acid	20.45	23.8
C18:3n-3c	(all-Cis-9,12,15) Methyl linoleate	α-linolenic acid	21.56	17.6
C20:0	Methyl arachidate	Arachidic acid	21.95	5.9
C20:1n-9c	(Cis-11) Methyl eicosanoate	Gondoic acid	23.00	15.0
C20:2n-6c	(all-Cis-11,14) Methyl eicosadienoate	Eicosadienoic acid	25.33	31.8
C21:0	Methyl heneicosanoate	Heneicosylic acid	25.46	1.8
C20:3n-6c	(all-Cis-8,11,14) Methyl eicosatrienoate	dihomo-γ-linolenic acid	26.63	18.6
C20:4n-6c	(all-Cis-5,8,11,14) Methyl arachidonate	Arachidonic acid	27.40	15.8
C20:3n-3c	(all-Cis-11,14,17) Methyl eicosatrienoate	Dihomolinolenic	27.47	1.5
C22:0	Methyl behenate	Behenic acid	27.91	10.2
C22:1n-9c	(Cis-13) Methyl erucate	Erucic acid	28.41	11.6
C20:5n-3c	(all-Cis-5,8,11,14,17) Methyl eicosapentaenoate	Timnodonic acid	28.97	14.6
C22:2n-6c	(all-Cis-13,16) Methyl docosadienoate	Docosadienoic acid	29.38	11.7
C23:0	Methyl tricosanoate	Tricosylic acid	29.50	3.5
C24:0	Methyl lignocerate	Tricosylic acid	30.73	34.6
C24:1n-9c	(Cis-15) Methyl nervonate	Nervonic acid	31.02	8.2
C22:6n-3c	(all Cis-4,7,10,13,16,19) Methyl docosahexanoate	Cervonic acid	31.78	23.8

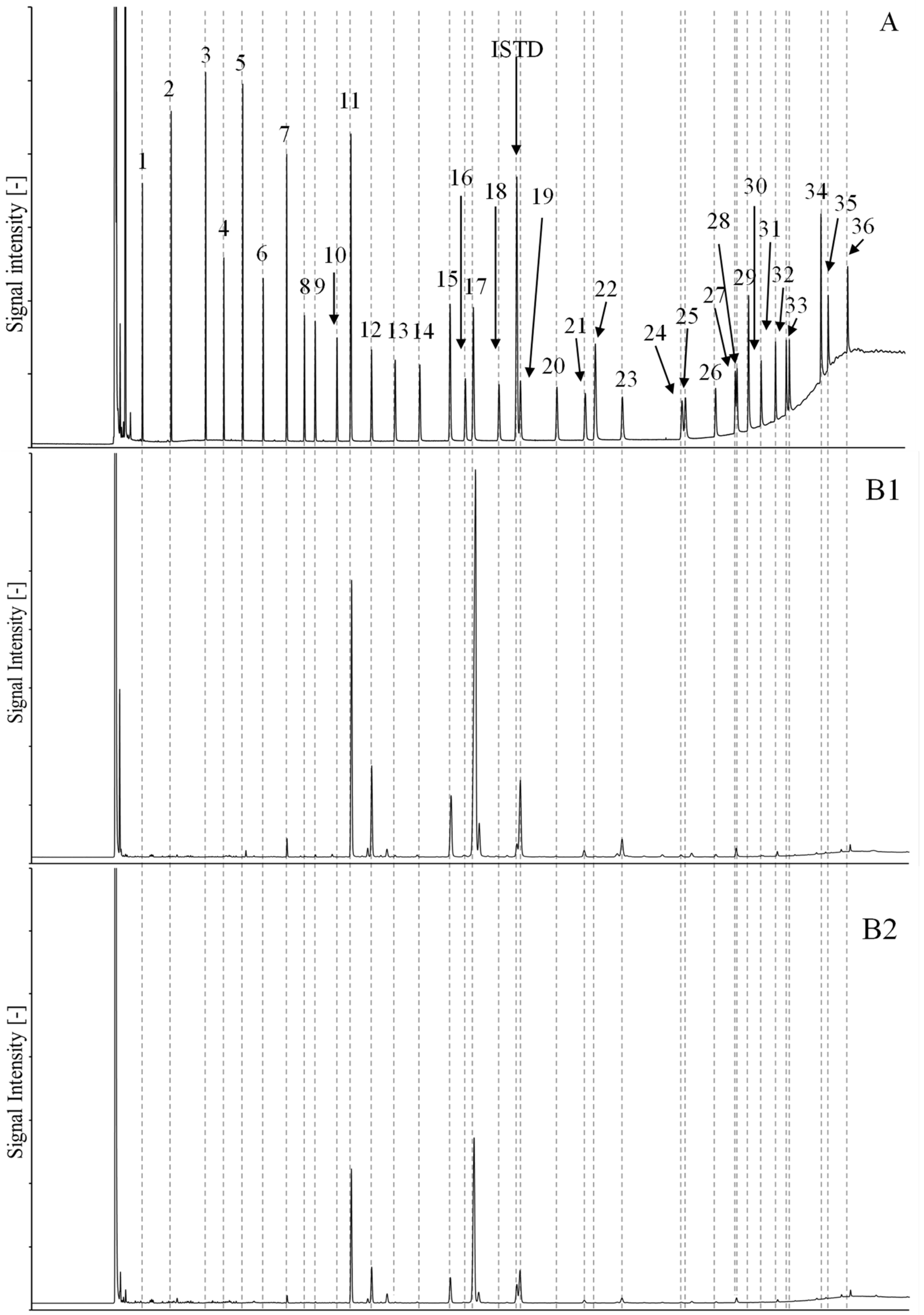

Figure S1. Chromatograms of FAME mixture (calibration level 4, spiked with 100 µg/mL C19:0ME) (**A**), small intestinal digesta of baked carp meal: *TFA method* (**B1**), *EFA method* (see the text for details) (**B2**). Numbers in the figure correspond to the peak numbers shown in Table 2.
